# An “Axiom *Cajanus* SNP Array” based high density genetic map and QTL mapping for high-selfing flower and seed quality traits in pigeonpea

**DOI:** 10.1186/s12864-019-5595-3

**Published:** 2019-03-21

**Authors:** Pooja Yadav, K. B. Saxena, Anupama Hingane, C. V. Sameer Kumar, V. S. Kandalkar, Rajeev K. Varshney, Rachit K. Saxena

**Affiliations:** 1 International Crops Research Institute for the Semi-Arid Tropics (ICRISAT), Patancheru, 502 324 India; 2 Department of Genetics and Plant Breeding, Rajmata Vijayaraje Scindia Krishi Vishwa Vidyalaya (RVSKVV), Gwalior, 474 002 India

**Keywords:** Cleistogamous flower, Selfing, Mapping, Shriveled seed, Axiom *Cajanus* SNP Array, QTLs

## Abstract

**Background:**

Pigeonpea has considerable extent of insect-aided natural out-crossing that impedes genetic purity of seeds. Pre-anthesis cleistogamy in pigeonpea promotes self-pollination which helps in maintaining genetic purity. The cleistogamous flowers are linked with shriveled seeds, an undesirable trait from variety adoption point of view, and breeding using genomics tools can help in overcoming this constraint. Therefore, in order to identify genomic regions governing these target traits, one recombinant inbred line (RIL) population was developed using contrasting parents (ICPL 99010 and ICP 5529) for flower shape and shriveled seeds. The RILs were phenotyped for two years and genotyped using the Axiom *Cajanus* SNP Array.

**Results:**

Out of the 56,512 unique sequence variations on the array, the mapping population showed 8634 single nucleotide polymorphism (SNPs) segregating across the genome. These data facilitated generation of a high density genetic map covering 6818 SNPs in 974 cM with an average inter-marker distance of 0.1 cM, which is the lowest amongst all pigeonpea genetic maps reported. Quantitative trait loci (QTL) analysis using this genetic map and phenotyping data identified 5 QTLs associated with cleistogamous flower, 3 QTLs for shriveled seed and 1 QTL for seed size. The phenotypic variance explained by these QTLs ranged from 9.1 to 50.6%. A consistent QTL “*qCl3.2*” was identified for cleistogamous flower on CcLG03 covering a span of 42 kb in the pigeonpea genome. Epistatic QTLs were also identified for cleistogamous flower and shriveled seed traits.

**Conclusion:**

Identified QTLs and genomic interactions for cleistogamous flower, shriveled seed and seed size will help in incorporating the required floral architecture in pigeonpea varieties/lines. Besides, it will also be useful in understanding the molecular mechanisms, and map-based gene cloning for the target traits.

**Electronic supplementary material:**

The online version of this article (10.1186/s12864-019-5595-3) contains supplementary material, which is available to authorized users.

## Background

Worldwide legumes are recognized for their high protein seeds and various soil improvement properties, as they occupy 5.8% of the total arable land [1]. For those living in the tropics and sub-tropics and earning their livelihoods with subsistence agriculture, pigeonpea [*Cajanus cajan* (L.) Millsp.] is an important legume crop. Globally pigeonpea is cultivated on 5.41 m ha with a production of 4.49 m t [2] in parts of Asia, Africa, Latin America, and Caribbean islands.

Most legume species are highly self-pollinated but there are some exceptions and pigeonpea is one of them with average insect-aided natural cross-pollination up to 40% [3]. Although the natural out-crossing of pigeonpea has been successfully used in breeding hybrids [4], it causes serious inefficiencies in pure line and maintenance breeding programs. There are several examples where the key varietal traits such as productivity, disease resistance, and seed quality were lost due to uncontrolled pollination of released cultivars [5, 6].

**Fig. 1:**
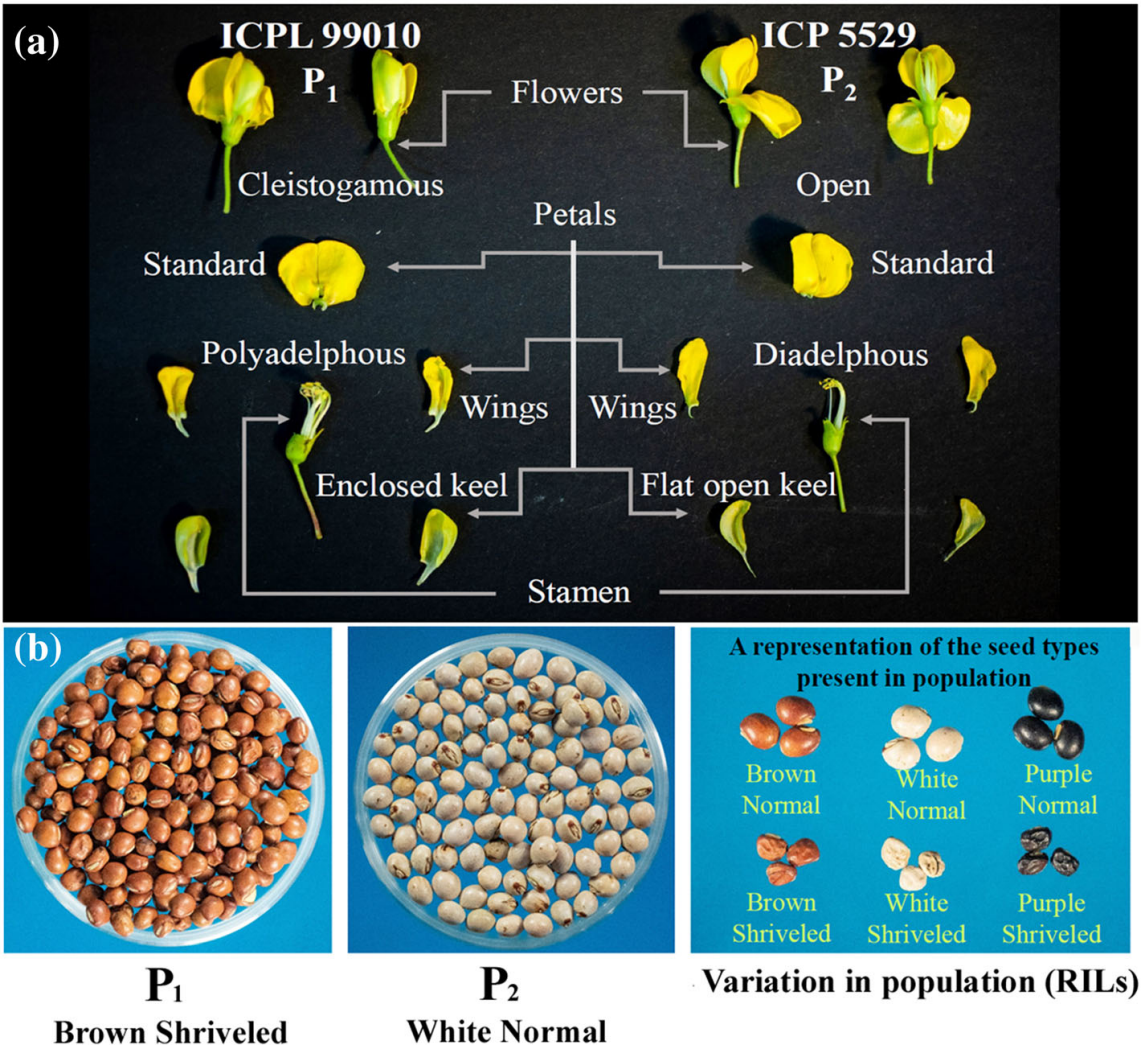
Diagrammatic representation of the contrasting features in crossing parents (P_1_: ICPL 99010 and P_2_: ICP 5529) of mapping population. **a** Dissection of cleistogamous flower in P_1_ and open flower in P_2_ to present difference in aestivation and stamen structure. **b** First from the left i.e. brown shriveled seeds in P_1_, second is white normal seed in P_2_ and extreme right section is representing seeds variation in the RILs

For a long time pigeonpea breeders were on the lookout for a genetic solution for this constraint and the success was achieved when Saxena et al. (1992) selected a unique recombinant within the segregating population of an inter-specific cross between *C. cajan* and *C. lineatus* [7]. This transgressive segregant, designated as “pre-anthesis cleistogamous flower”, was characterized by delayed flower opening and modified floral morphology and anther configuration. In such unique flowers, the keel, standard and wing petals are wrapped (Fig. 1a). Also in these flowers, unlike typical pigeonpea flowers with diadelphous (9 + 1) anther configuration, all the 10 anthers are individually attached to the base. Saxena et al. [8] demonstrated that in the lines with cleistogamous flowers the natural out-crossing under field conditions was around 2%, as compared to over 30% in the genotypes with normal flowers. Unfortunately, the pre-anthesis cleistogamous flower trait was found to be strongly linked to undesirable traits such as shriveled and small seeds (Fig. 1b). Such genetic linkages adversely affect the breeding of marketable pigeonpea cultivars with high self-pollinating habit. This constraint, however, can be overcome through the integration of modern genomics technologies in pure line breeding programs.

In the last decade there have been significant developments in pigeonpea genomics including development of molecular markers e.g. simple sequence repeats (SSRs) [9–12] single nucleotide polymorphism (SNP) [13], high density genotyping platforms [13–15] genetic maps [10, 13, 16, 17], transcriptome assemblies [12, 18, 19], gene expression atlas [20], reference genome [21] and whole genome re-sequencing data for hundreds of pigeonpea lines [22, 23] have become available. These resources have been used in identification of genomic segments or quantitative trait loci (QTL) associated with economical important traits for pigeonpea improvement [24–27]. However, genomic segments/molecular markers associated with cleistogamous flower and shriveled seed in pigeonpea have not been identified till now. Therefore, the present study has been planned to use one recombinant inbred line (RIL) population developed for cleistogamous flower and shriveled seed traits. The high density genotyping with Axiom *Cajanus* SNP Array [28] and phenotyping of RILs have provided genomic segments associated with cleistogamous flower and shriveled seed traits. Further, we have also identified a number of interactions in the genomic segments responsible for above mentioned traits. These results will be of help in designing the crop improvement strategies for understanding/transferring the cleistogamous flower in released/target varieties with desirable seed size and shape following genomics-assisted breeding (GAB) approaches.

## Results

### Phenotyping of target traits

In the present study a high-selfing cleistogamous line ICPL 99010 was crossed with an adapted pigeonpea inbred line ICP 5529 and a mapping population of 80 RILs was developed. The phenotypic data on cleistogamous flowers and shriveled seeds were collected for two years on 80 RILs derived from this cross in F_6_ and F_7_ generations. In year 1, 35 RILs showed cleistogamous flowers while 45 RILs had open flowers. To confirm the traits stability in year 2, 10 to 12 individual plants from each RIL were phenotyped in progeny to row manner. Out of 80 RILs (880 plants), 76 RILs (832 plants) had no intra-progeny segregation or true types to the first year phenotype (cleistogamous flowers: 33 RILs and open flowers: 43 RILs), whereas, 48 plants representing 4 RILs showed segregation for cleistogamous and open flowers. The segregation pattern for cleistogamous and open flowers in RILs have indicated the involvement of single gene with *P* value 0.26 and 0.25 in year 1 and year 2 respectively (Additional file 1: Table S1).

**Fig. 2:**
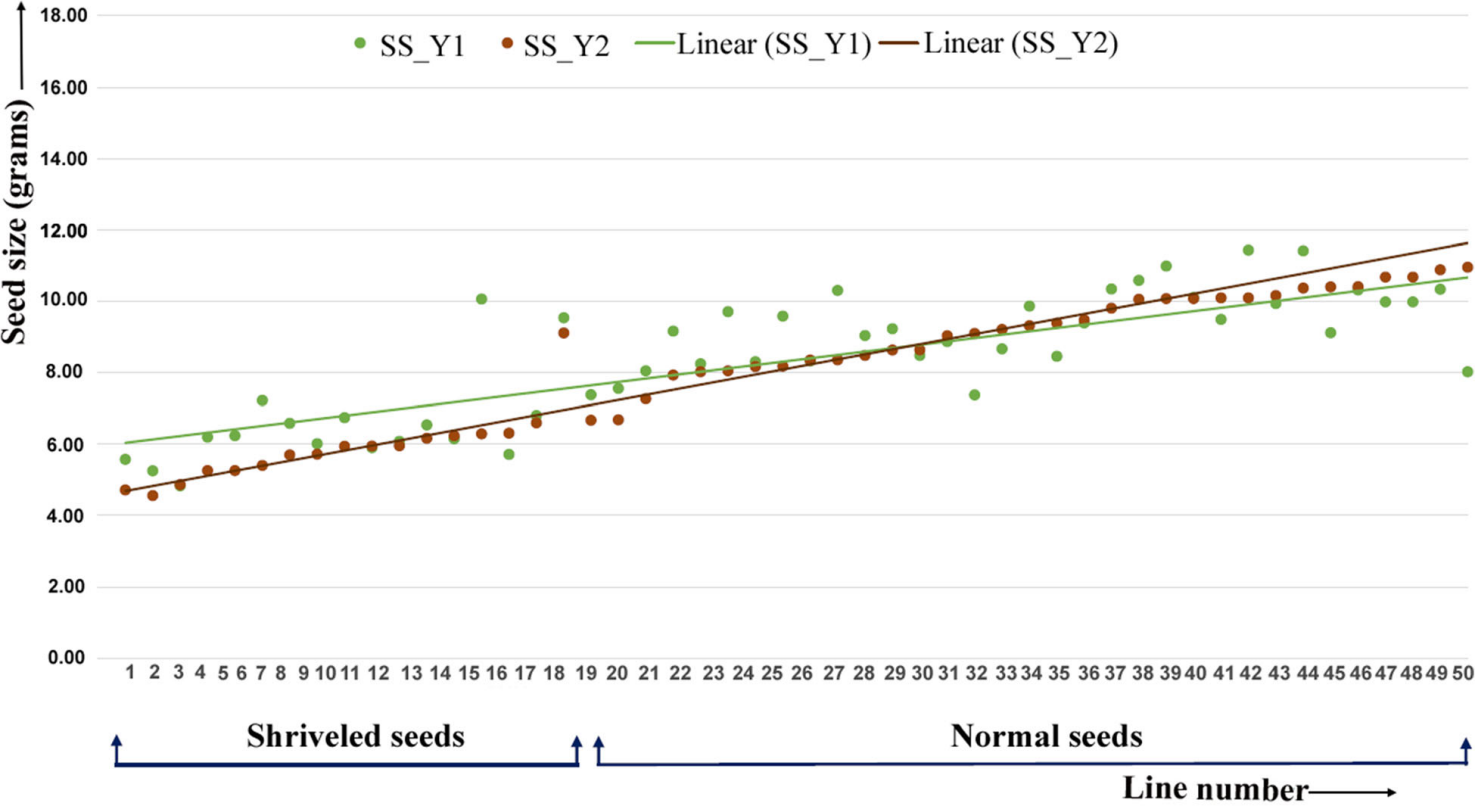
Scatter plot showing lower seed size in shriveled seeds compared to normal seeds. “Y-axis” represents the seed size (100 seed weight in grams) and “X-axis” shows line numbers

For second target trait i.e. shriveled/normal seeds, the phenotypic data were collected on all the harvested seeds from individual plants. In the year 1, 21 and 59 RILs possessed shriveled and normal seeds respectively. Similar to the cleistogamous flower trait in the year 2, seeds from individual plants were also phenotyped. Out of 80 RILs (880 plants), 61 RILs (684 plants) had no segregation or true types to the first year phenotype (shriveled seeds: 17 RILs and normal seeds: 44 RILs), whereas, 196 plants in 19 RILs showed segregation for shriveled and normal seeds. Phenotyping data on shriveled/normal seeds have shown possible control of two genes on this trait (*P* value 0.8 and 0.6 in year 1 and year 2 respectively) (Additional file 1: Table S1). In parallel, seed size data were also recorded in RILs for the year 1 and the year 2. In the year 1, the seed size ranged from 4.8 g to 11.4 g with an average of 8.5 g whereas in the year 2, it ranged from 4.6 g to 17.8 g with an average of 8.4 g. In order to see the possible correlation between seed size data with the shriveled and normal seeds, a scatter plot was formed with the RILs showing consistent behavior in both the years. In general, in both the years shriveled seeds showed lesser seed size as compared to normal seeds (Fig. 2). In year 1 the average value of seed size in 16 RILs carrying shriveled seeds was 6 .4g and in 37 RILs carrying normal seeds was 9 .4g. Almost similar observations were made in year 2, where average value of seed size in 16 RILs carrying shriveled seeds was 5 .7g and in 37 RILs carrying normal seeds was 9 .5g.

### High density genetic map with 6 .8K SNPs

**Fig. 3:**
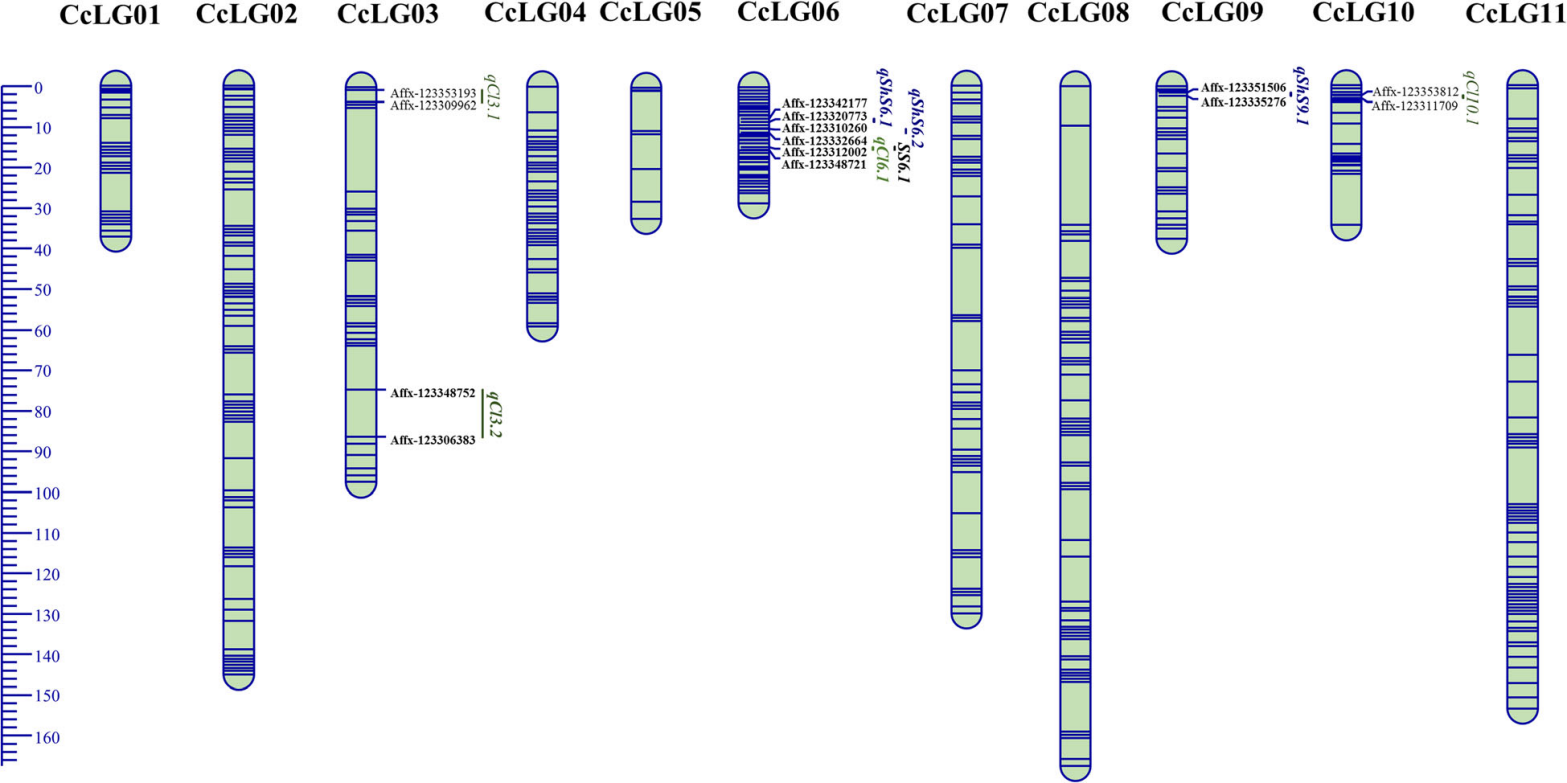
6 .8K high density genetic map with QTLs for target trait. QTL names in green color are for cleistogamous flower; QTL names in blue color are for shriveled seed; QTL names in black color for seed size

High quality DNA could be isolated from 72 RILs from a total of 80 RILs. These 72 RILs were used for high density genotyping using Axiom *Cajanus* SNP Array. Out of 56,512 SNPs placed on Axiom *Cajanus* SNP Array, the genotyping data on 72 RILs and parents were generated for 55,748 SNPs (Table 1). From these SNPs, 11,478 SNPs were polymorphic and 44,270 SNPs were monomorphic between parents. Further, polymorphic SNPs showing heterozygous alleles (2830 SNPs) in any of the parents and 14 duplicated SNPs in Axiom *Cajanus* SNP Array (placed two times in array due to their importance) [28] were removed from further analysis. As a result, a total of 8634 non redundant SNPs with homozygous alleles and polymorphic between parents were considered for the construction of genetic map (Table 1). Genotyping data obtained for all 8634 SNPs on RILs were tested for the Mendelian/non-Mendelian segregation pattern. The largest group i.e. 5733 SNPs were categorized into *P* values ranging from 0.00–0.05. While the smallest group of 264 SNPs were falling into *P* values ranging from 0.06–0.10. The other two marker groups contained 1384 and 1253 SNPs with* P* values of 0.11–0.50 and 0.51–1.00 respectively. Genotyping data for 8634 polymorphic SNPs generated on 72 RILs were used for genetic map construction. Eleven *Cajanus cajan* linkage groups (CcLGs: CcLG01–CcLG11) were generated using an LOD (logarithm of the odds) threshold value of 5. A total of 1816 SNPs that failed to be assigned to these CcLGs were not incorporated in further genetic mapping. As a result, 6818 SNPs could be mapped on 11 CcLGs covering 974 cM (Table 1). CcLG05 was the shortest with 248 SNPs mapped with 34 cM distance. The longest was CcLG11 with 1258 mapped SNPs covering a distance of 153 cM. Highest marker density was observed for CcLG01, which had 14.9 markers per cM, whereas the lowest marker density was observed for CcLG07 with 4.0 markers per cM. Overall, the genetic map had 7 markers per cM (Table 1, Fig. 3, Additional file 2: Table S2).

**Table 1:** Linkage group wise distribution and summary on SNPs used for genetic map construction

Linkage groups	SNPs on Axiom *Cajanus SNP* Array	Data generated	SNPs polymorphic in/for parents	Homozygous polymorphic SNPs	SNPs used for constructing genetic map	SNPs mapped	% Mapped	Length (cM)	Average marker interval (cM)	SNPs/cM
CcLG01	4638	4585	861	629	629	577	91.7	39	0.1	14.9
CcLG02	8506	8425	1311	1045	1043	882	84.6	146	0.2	6.0
CcLG03	5869	5773	1250	945	944	493	52.2	98	0.2	5.0
CcLG04	4134	4093	830	627	627	608	97.0	59	0.1	10.3
CcLG05	922	904	335	251	251	248	98.8	34	0.1	7.3
CcLG06	4784	4693	943	750	747	659	88.2	53	0.1	12.4
CcLG07	4321	4269	813	632	629	526	83.6	130	0.2	4.0
CcLG08	5178	5099	1093	793	791	759	96.0	167	0.2	4.5
CcLG09	3615	3578	780	527	527	350	66.4	39	0.1	9.1
CcLG10	5928	5834	1192	742	742	458	61.7	56	0.1	8.1
CcLG11	8617	8495	2070	1707	1704	1258	73.8	153	0.1	8.2
Total/ across genome	56,512	55,748	11,478	8648	8634	6818	79.0	974	0.1	7.0

### QTLs for target traits

Phenotypic data together with SNP genotyping data were used for QTL analysis using composite interval mapping (CIM). Based on the phenotypic variance explained (PVE), identified QTLs were classified as major (≥10% PVE) and minor QTLs (< 10% PVE). The identified QTLs were also classified as consistent QTLs (appeared in more than one year). For each trait details on QTLs identified have been explained below.

### QTLs for cleistogamous flower

A total of five QTLs (four in year 1 and one in year 2) were identified for cleistogamous flower. In year 1, two major QTLs (*qCl3.2* and *qCl6.1*) on CcLG03 and CcLG06 and two minor QTLs (one each on CcLG03 and CcLG10) were detected (Table 2). The *"qCl3.2"* QTL flanked by Affx-123,348,752 and Affx-123,306,383 markers has shown 38% PVE. Another major QTL i.e. *"qCl6.1"* flanked by Affx-123,312,002 and Affx-123,348,721 markers has shown 19.7% PVE. Whereas, in the year 2 only one major QTL flanked by Affx-123,348,752 and Affx-123,306,383 markers with 50.6% PVE was identified (Table 2). The major QTL “*qCl3.2*” flanked by Affx-123,348,752 and Affx-123,306,383 markers was also classified as consistent QTL for cleistogamous flower. Flanking markers (positions in the draft genome) of the consistent QTL “*qCl3.2*” were used to retrieve the gene information from the draft genome [21]. As a result, 20 genes were found in 42 Kb genomic region covered by "*qCl3.2"*. Out of 20 genes, 5 were uncharacterized and for two genes with unknown function (Additional file 3: Table S3). Functional annotation of remaining 13 genes have shown their role in biological process such as cell wall synthesis, protein transport, cell signaling and in defense mechanism such as response to heat shock etc. Further, above mentioned 20 genes in *"qCl3.2"* can be considered as a starting point in identification of causal genes responsible for cleistogamous flower in pigeonpea. A number of approaches can be used in future for this purpose such as region specific deep sequencing or whole genome resequencing based fine mapping, functional studies based on transcriptome, etc.

**Table 2:** QTLs identified for target traits in RILs developed from ICPL 99010 × ICP 5529

QTL ID	Trait name	CcLG	Position (cM)	Left marker	Right marker	LOD	PVE(%)	Add	Left CI	Right CI
*qCl3.1*	Cleisto_Y1	03	1	Affx-123,353,193	Affx-123,309,962	32.0	9.1	0.2	0.0	1.5
*qCl3.2*	Cleisto_Y1	03	85	Affx-123,348,752	Affx-123,306,383	26.5	38.0	−0.3	82.5	85.5
*qCl6.1*	Cleisto_Y1	06	15	Affx-123,312,002	Affx-123,348,721	7.2	19.7	−0.2	14.5	15.5
*qCl10.1*	Cleisto_Y1	10	3	Affx-123,353,812	Affx-123,311,709	38.1	9.1	0.2	2.5	3.5
*qCl3.2*	Cleisto_Y2	03	82	Affx-123,348,752	Affx-123,306,383	466.2	50.6	−0.5	79.5	84.5
*qShS6.2*	ShS_Y1	06	11	Affx-123,310,260	Affx-123,332,664	6.0	33.3	−0.3	10.5	11.5
*qShS6.1*	ShS_Y2	06	8	Affx-123,342,177	Affx-123,320,773	9.0	37.2	−0.3	6.5	8.5
*qShS9.1*	ShS_Y2	09	2	Affx-123,351,506	Affx-123,335,276	3.4	11.8	0.2	1.5	3.5
*qSS6.1*	SS_Y2	06	15	Affx-123,312,002	Affx-123,348,721	5.1	29.5	−1.4	14.5	15.5
*qSS6.1*	SS_Y1 + Y2	06	15	Affx-123,312,002	Affx-123,348,721	4.6	33.9	−1.2	14.5	15.5

### QTLs for shriveled seed

There were three major QTLs (one appeared in the year 1 and two appeared in the year 2) identified for shriveled seed on CcLG06 and CcLG09. In the year 1, the major QTL “*qShS6.2*” on CcLG06 flanked by Affx-123,310,260 and Affx-123,332,664 has shown 33.3% PVE. Whereas, in the year 2, two major QTLs one each on CcLG06 (*qShS6.1*) and CcLG09 (*qShS9.1*) were identified. The QTL “*qShS6.1*” flanked by Affx-123,342,177 and Affx-123,320,773 explained phenotypic variation of 37.2% and QTL “*qShS9.1*” flanked by Affx-123,351,506 and Affx-123,335,276 had 11.8% PVE (Table 2). Though major QTLs namely “*qShS6.1*” and “*qShS6.2*” were closely located on CcLG06 (at 3 cM distance) appeared in the year 1 and 2, respectively but not considered as consistent QTL. A total of 113 genes were found in the above mentioned QTL regions. The QTLs “*qShS6.1*” (21 kb), “*qShS6.2*” (22 kb) and “*qShS9.1*” (16 kb) contained 13, 96 and 4 genes respectively (Additional file 4: Table S4).

### QTLs for seed size

Two major QTLs (one each appeared in the year 2 and in pooled analysis) were detected for seed size on CcLG06 (Table 2). In the year 1, though few minor QTLs with very low PVE were detected but no major QTL identified. In the year 2, one major QTL “*qSS6.1*” flanked by Affx-123,312,002 and Affx-123,348,721 markers with 29.5% PVE was identified. The pooled analysis of year 1 and 2 data has also detected “*qSS6.1*” with 33.9% PVE.

### Epistatic QTLs associated with target traits

**Fig. 4:**
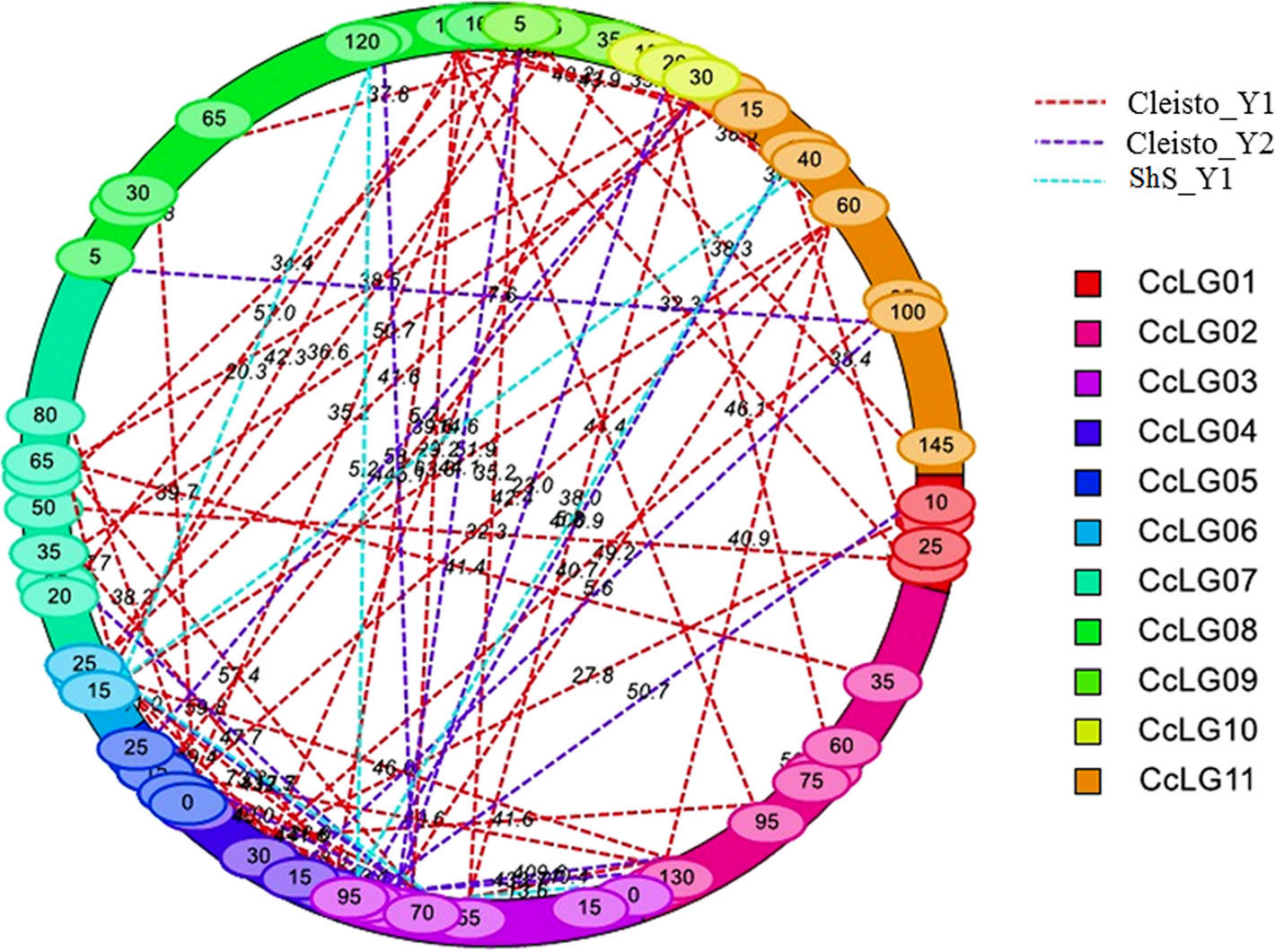
A schematic illustration of the epistatic QTLs for cleistogamous flower and shriveled seed traits. Different colors in the ring represent 11 CcLGs. The number in ovals on ring indicating marker positions on CcLGs. Dash lines are representing epistatic interactions between loci. Numbers on dash lines indicating the LOD scores of additive × additive effects

The epistatic interactions for all three traits were also analyzed. As a result, 64 pairwise QTLs were detected across all the CcLGs (Fig. 4, Additional file 5: Table S5). These pairwise QTLs have shown the PVE in the range of 1.2 to 8.5% with LOD > 5. There were 59 epistatic QTLs involved with cleistogamous flower whereas, 5 epistatic QTLs associated with shriveled seed. The genetic variance caused by additive × additive epistasis was ranging from − 0.3 to + 0.5 in 64 pairwise QTLs. There were 13 different epistatic interactions (4 in year 1 and 9 in year 2) found for the consistent QTL “*qCl3.2”* for cleistogamous flower with similar PVE of 5.9%. The QTL “*qCl6.1”* for cleistogamous flower showed 8 epistatic interactions in which 5 epistatic QTL pairs were detected for cleistogamous flower (4 in year 1 with 3.7% PVE and 1 in year 2 with 5.9% PVE) whereas 3 epistatic QTL pairs were found for shriveled seed with 8.5% PVE (Table 3). There was no significant epistatic interaction found for seed size.

**Table 3:** Epistatic interactions identified for cleistogamous flower and shriveled seeds across pigeonpea genome

Trait	CcLG	Total interactions	^a^CcLGs in interaction	PVE range
Cleistogamous flower	CcLG01	5	**3**, 4, 7, 8, 11	1.2–5.9
	CcLG02	9	**3**, 4, 5, 6, 7, 8, 10, 11	1.2–5.9
	CcLG03	16	**4, 5, 6, 7, 8, 9, 10, 11**	1.2–5.9
	CcLG04	7	5, 6, 7, 8, 9, 11	1.2–3.7
	CcLG05	7	6, 7, 8, 9, 10, 11	1.2–1.8
	CcLG06	5	7, 8, 9, 10, 11	1.2–3.7
	CcLG07	4	8, 9, 10, 11	1.2
	CcLG08	4	9, 10, 11	1.2–2.5
	CcLG09	1	11	1.2
	CcLG10	1	11	1.2
Shriveled seed	CcLG03	3	**6, 8, 11**	6.0–8.5
	CcLG06	2	**8, 11**	8.5

^a^CcLGs in bold show a PVE % of ≥5

## Discussion

Pigeonpea improvement activities over the last sixty years have led to the release of over 100 varieties through pedigree breeding http://iipr.res.in/pdf/iipr_piegeonpea.pdf In these cultivars, a number of advancements were made in terms of crop adaptation patterns, reduction of crop duration, enhanced diseases resistance etc. These traits have helped in increasing the area but sustaining these gains is becoming difficult due to varietal contamination caused by natural cross-pollination. The incorporation of pre-anthesis cleistogamy [29] in future pigeonpea cultivars can help in overcoming this menace. Although, some efforts were made in the past to incorporate this trait but so far no success has been achieved in breeding high yielding high-selfing cultivars due to the difficulties posed by its adverse association with unwanted seed traits. Inheritance studies of cleistogamous flower trait in pigeonpea [29], durum wheat [30] and rice [31] revealed its single gene control. Whereas, in soybean [32] and barley [33] two or more genes showing epistatic interactions governed this trait. The development of a dense genetic map and genomic regions associated with cleistogamous flower, shriveled seed and seed size is an important event in pigeonpea breeding which can lead to a breakthrough in breeding the desired genotypes.

A genetic map containing 6818 SNPs with a map distance of 974 cM was constructed using Axiom *Cajanus* SNP array. Number of markers mapped in the present study was higher than the earlier genetic maps in pigeonpea [10, 13, 16, 17, 25–27, 34, 35]. The average inter-marker distance observed (0.1 cM) was the lowest in all the genetic mapping studies conducted in pigeonpea so far [10, 13, 16, 17, 25–27, 34, 35], which indicates better saturation of available genetic map. The present genetic map has shown only 2 large gaps one each on CcLG03 and CcLG08 with marker intervals > 20 cM. Therefore, the developed genetic map in the present study will be an important resource for not only QTL identification and understanding the association between cleistogamous flower with shriveled seed trait but also in QTL cloning and identification of candidate genes.

QTL mapping for cleistogamous flower, shriveled seed and seed size have revealed 10 significant QTLs with 8 of these QTLs showing major effects with PVE of more than 10%. The identified QTLs in the present study are novel as this is the first report of mapping QTLs for above mentioned traits in pigeonpea. However, only one consistent QTL for cleistogamous flower and no consistent QTLs for shriveled seeds and seed size were identified. This might be due to the presence of environment interactions over the years coupled with small population size. It is interesting to note that we found 5 QTLs on CcLG06 with significant effects for cleistogamous flower, shriveled seeds and seed size.

For pigeonpea, the association between any modification to the flower structure and deformed or shriveled seed shape has been commonly observed, including in the present study. Moreover, the genomic segments or QTLs for these traits have not been investigated earlier and remain unknown. The existence of five major QTLs (*qCl3.2*, *qCl6.1*, *qShS6.1*, *qShS6.2* and *qSS6.1*) with opposite effects for cleistogamous flower, shriveled seeds and seed size in the current study provides an ideal example to test above mentioned hypothesis at the genome level. This is also the first study that provides genomic evidences to clearly demonstrate the presence of linkage drag among cleistogamous flower, shriveled seeds and seed size in pigeonpea. It is also important to mention here that shriveled seed may have a confounding effect on seed size as also seen in the regression between the two traits; hence seed size should be mapped in a population or sub-population with normal seeds only. In this study, the presence of only additive × additive epistatic interactions reaffirms the fact that the trait was governed by one or two major genes. Also the range of PVE was very less for cleistogamous flower interactions but was high for the major consistent QTL which suggested that there were not many genomic regions governing this trait and the major contribution for cleistogamous flower was attributed to “*qCl3.2*”. Therefore, a strategy is required to introgression of *"qCl3.2"* through GAB for transferring cleistogamous flower in elite cultivars without decreasing the seed size and shriveled seeds.

## Methods

### Mapping population

The experimental material consisted of 80 RILs derived from a cross between ICPL 99010 and ICP 5529. The female parent (ICPL 99010) contains cleistogamous flowers and shriveled seeds, whereas, the male parent (ICP 5529) had open flowers and normal seeds (Fig. 1). The F_1_s produced were advanced till F_6_ generation by using single seed descent method. The population was advanced with 75 cm distance between rows and 30 cm distance between plants in a 4 m long row following the recommended agronomic practices as mentioned in Obala et al. [36].

### Phenotyping

Phenotyping of RILs for cleistogamous flower and shriveled seed traits was carried out in two cropping seasons (2016–17 and 2017–18). The flowers from each RIL were phenotyped for cleistogamous and open flower based on visual assessment in the field three times in a day viz. morning (0700 h), afternoon (1300 h) and evening (1800 h). The observations on cleistogamous and open flower were taken on at least 10 flowers representing different parts of the single plant. The phenotypic observation divided flowers into two categories based on pattern of arrangement of petals and fused or non-fused anthers. The cleistogamous flowers remained closed while the open flowers gradually exposed its stamen and carpel when buds bloomed into flowers. On the contrary, cleistogamous flowers were observed to be enwrapped throughout the flowering season viz. initiation of flowering, at 50% flowering, at 75% flowering till the pod formation. In year 1 (2016–17) single plant representing individual RIL was phenotyped, whereas, in second year (2017–18) 10–12 plants were phenotyped from each RIL. Thus a total of 880 plants representing 80 RILs were phenotyped for cleistogamous flower in year 2. Harvested seeds from all above mentioned plants representing 80 RILs were also phenotyped for shriveled or normal shape and seed size in both the years.

### DNA isolation and SNP genotyping

Genomic DNA was isolated from three to four young leaves of individual plants of each RIL, using a NucleoSpin Plant II kit (Macherey-Nagel, Düren, Germany). The quality of DNA was checked on 0.8% agarose gel and DNA quantity assessed on Qubit® 2.0 Fluorometer (Thermo Fisher Scientific Inc., USA). The high density genotyping on RILs was performed through 56 K Axiom *Cajanus* SNP Array as described in Saxena et al. (2018) [28]. This Axiom *Cajanus* SNP Array has been developed recently using WGRS data on elite breeding lines and filtering sequence variations through Axiom GTv1 algorithm [28].

### Genetic map construction

The maternal and paternal alleles were marked ‘2’ and ‘0’ while the missing values were marked ‘-1’. The linkage analysis was done by comparing SNPs with expected segregation ratio of 1:1 using χ^2^ test. The SNPs were sorted and grouped into different CcLGs. The SNPs showing polymorphism between parental genotypes were selected for construction of genetic map. The MAP model of QTL IciMapping software version 4.1 (www.isbreeding.net) was used for construction of genetic map [37]. QTL IciMapping was based on inclusive composite interval mapping (ICIM) method [38, 39]. Kosambi mapping function was used to estimate the map distances (cM) from recombination frequencies [40]. There were three steps in building a linkage map using ICIMapping viz. grouping, ordering and rippling. Grouping in QTL IciMapping was done based on regression mapping algorithm with a threshold LOD score of 5. Ordering algorithm of ‘nnTwoOpt’ and the rippling criteria SARF (sum of adjacent recombination frequencies) were used with a window size of 5 SNPs.

### Quantitative trait locus (QTL) analysis

The phenotypic data together with the genotyping data were used for QTL analysis for target traits using BIP model of QTL IciMapping version 4.1 [37]. The main effect QTLs were determined by ICIM-ADD with a default LOD threshold value of 2.5. The nomenclature of QTLs include “*q*” for quantitative trait (distinguished from major gene) followed by trait code (“*Cl*” for cleistogamous flower, “*ShS”* for shriveled seeds and “*SS”* for seed size) with chromosome/linkage group number and chronological order of QTL for that trait on the chromosome/ linkage group separated by a dot. The epistatic effect QTLs were determined by ICIM-EPI in BIP model of QTL IciMapping v4.1. with a LOD threshold value of 5 [37, 41].

## Conclusions

A high density genetic map was constructed using Axiom *Cajanus* SNP Array. Based on genetic map and phenotypic data, one consistent QTL for pre-anthesis cleistogamy was identified on CcLG03. This QTLs can be deployed in GAB for development of pigeonpea genotypes with cleistogamous flowers and acceptable seed size and shape.

## Additional files

Additional file 1: **Table S1.** Chi-square distribution for cleistogamous flower and shriveled seed traits in RILs. (XLSX 10 kb)

Additional file 2: **Table S2.** SNP ID, map position, genotyping data and *P* value for 6 .8K SNPs used to construct genetic map in RILs developed from ICPL 99010 × ICP 5529 (XLSX 1988 kb)

Additional file 3: **Table S3.** List of genes and their functions in the 42 kb region of the consistent QTL “*qCl3.2*” for cleistogamous flower. (XLSX 10 kb)

Additional file 4: **Table S4.** List of genes and their functions in QTL regions for shriveled seeds trait. (XLSX 16 kb)

Additional file 5: **Table S5.** Epistatic QTLs among two years’ data for cleistogamous flower and shriveled seed in pigeonpea. (XLSX 15 kb)

## Abbreviations

CcLG: *Cajanus cajan* linkage group
CI: Confidence interval
CIM: Composite interval mapping
cM: centimorgan
DNA: Deoxyribonucleic acid
GAB: Genomics-assisted breeding
ICIM: Inclusive composite interval mapping
LOD: Logarithm of the odds
PVE: Phenotypic variance explained
QTL: Quantitative trait loci
RIL: Recombinant inbred line
SARF: Sum of adjacent recombination frequencies
SNP: Single nucleotide polymorphism
SSR: Simple sequence repeat
